# Ventriculoperitoneal shunt malfunction diagnosis based on substance dilution

**DOI:** 10.1097/MD.0000000000026770

**Published:** 2021-08-06

**Authors:** Xinjie Fu, Yuhang Chen, Weike Duan, Haixin Yang, Jiulin Xu, Xiaobing Cheng, Hongri Zhang

**Affiliations:** Department of Neurosurgery, The First Affiliated Hospital, and College of Clinical Medicine of Henan University of Science and Technology, Luoyang, China.

**Keywords:** dilution, malfunction, puncture, sodium valproate, ventriculoperitoneal shunt

## Abstract

**Objective:**

Current methods for the diagnosis of ventriculoperitoneal (VP) shunt malfunction lack specific standards; therefore, it may be missed or misdiagnosed. Hence, providing a reliable diagnostic method will help improve the accuracy of preoperative decision-making. Therefore, the aim of the study was to provide a new method for the diagnosis of VP shunt malfunction.

**Methods:**

After in vitro testing, we enrolled a total of 12 patients with VP shunt malfunction. Before revision surgery, 0.1 mL of a 5% sodium valproate (SV) solution was injected into the reservoir; 0.1 mL of the cerebrospinal fluid (CSF) was withdrawn 20 minutes later from the reservoir to measure the SV concentration. The process was repeated on the seventh day after surgery and compared with the preoperative results.

**Results:**

The mean ± standard deviation preoperative SV concentration in the cerebrospinal fluid was greater than the postoperative concentration (5967.8 ± 1281.3 vs 391.1 ± 184.6 μg/mL, *P* = .001).

**Conclusion:**

The proposed method is a reliable, safe, and relatively simple alternative for the diagnosis of VP shunt malfunction and further provides a reference for treatment.

## 1 Introduction

Ventriculoperitoneal (VP) shunt malfunction is a common complication of shunt surgery.^[1–3]^ When computed tomography (CT) and magnetic resonance imaging (MRI) scans show an enlarged ventricular system, it can indicate a malfunction; in contrast, squeezing the reservoir and B-ultrasound examination can also help in the diagnosis.^[4,5]^ However, current methods lack specific standards and VP shunt malfunction may be missed or misdiagnosed. Once malfunction is diagnosed, shunt revision surgery is required. Therefore, providing a reliable method to diagnose VP shunt malfunction before surgery will help improve the accuracy of preoperative decision-making, which was the aim of this study.

## 2 Materials and methods

### 2.1 Participants

The study protocol was approved by the Ethics Committee of the First Affiliated Hospital of Henan University of Science and Technology (Luoyang, Henan, China), and written informed consent was obtained from all the participants. After in vitro testing, 12 patients diagnosed with VP shunt malfunction were enrolled in this study. Patients and their family members were informed of the relevant accidents and complications that might occur during the experimental procedure. Written informed consent was obtained from the family members before enrollment in this study. The patients included 7 men and 5 women aged between 21 and 64 years, with a mean ± standard deviation age of 43.7 ± 8.2 years. All of them had secondary hydrocephalus, including 5 with subarachnoid hemorrhage, 1 with craniopharyngioma, 4 with craniocerebral trauma, 1 with cholesteatoma, and 1 with cerebral infarction. The measurements were performed before shunt revision and on the seventh day after revision. We defined preoperative patient measurements as the malfunction group and postoperative patient measurements as the control group for comparison.

### 2.2 In vitro experiment

**Figure 1:**
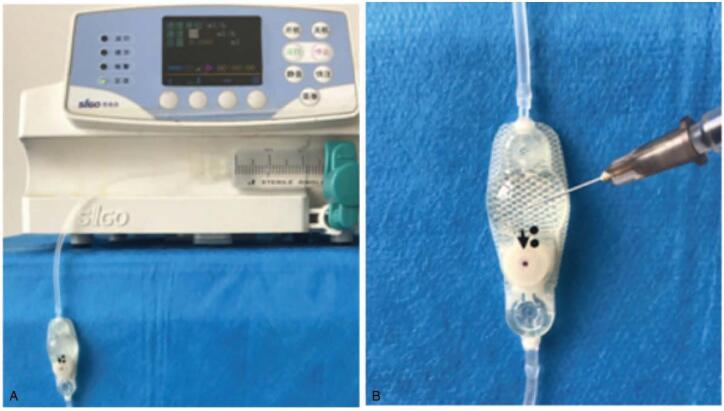
Schematic diagram of the in vitro experiment. (A) Introduction of the simulated cerebrospinal fluid shunt to set different flow rates. (B) Puncture site of the reservoir.

The shunt device was connected to a microinfusion pump and the valve vertically fixed. First, physiological saline was passed through the Medtronic PS Medical Delta valve (Medtronic, Minneapolis, MN) at different velocities controlled by the pump (Fig. 1A). Thereafter, 0.1 mL of a 50 mg/mL sodium valproate (SV) physiological saline solution was injected into the reservoir, and 0.1 mL of the solution in the reservoir was siphoned 20 minutes later. The siphoned solution was diluted (1:15) in a physiological saline solution for determination of the SV concentration with an automatic chemiluminescence immunoassay analyzer (ADVIA Centaur XP, Siemens Healthcare Diagnostics Inc.) (Fig. 1B),^[6]^ which constructed a graph of the SV flow rate versus its concentration. Second, we chose a flow rate of 2.1 mL/h as an obstruction group and 10 mL/h as a control group. For each group, we performed 8 experiments and compared the test results.

### 2.3 In vivo experiment

**Figure 2:**
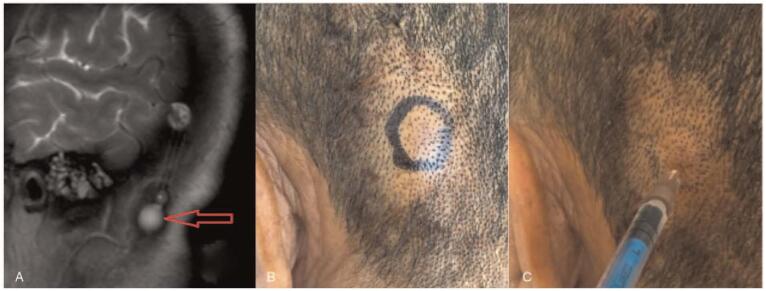
Schematic diagram of the in vivo experiment. (A) The red arrow in the magnetic resonance image marks the location of the reservoir. (B) Puncture site mark in patients in the obstruction group. (C) 0.1 mL of a physiological saline solution containing 5 mg of sodium valproate was injected into the reservoir through the skin.

In the examination of magnetic resonance image we can located the reservoir (Fig. 2A). All patients were placed in the prone position for 1 hour and instructed to assume a sitting position while the puncture site was labeled (Fig. 2B) and sterilized. Thereafter, 0.1 mL of a 50 mg/mL SV physiological saline solution was injected into the reservoir (Fig. 2C). After 20 minutes, 0.1 mL of cerebrospinal fluid (CSF) was siphoned from the reservoir and the SV concentration was measured as in the in vitro experiment.

### 2.4 Statistical analysis

SPSS version 26.0 software (IBM Corp., Armonk, New York, NY) was used for statistical analysis. The data of the 2 groups were compared using the paired *t* test. Statistical significance was set at *P* = .001.

## 3 Results

### 3.1 In vitro experiment

**Figure 3:**
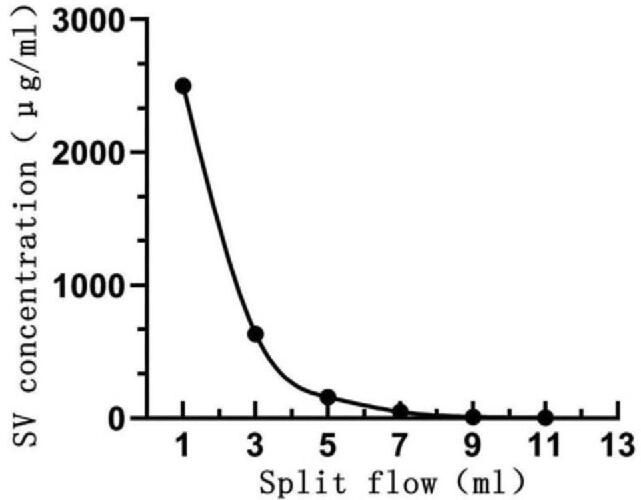
Schematic diagram of in vitro experimental results. The sodium valproate concentration decreases with an increasing saline flow rate. SV  = sodium valproate.

**Figure 4:**
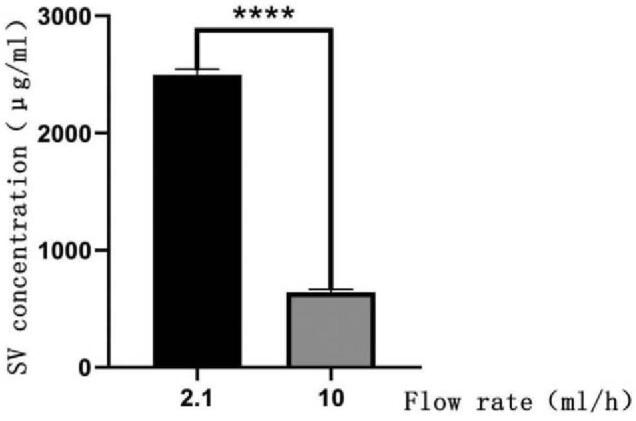
Schematic diagram of in vitro experimental results (n = 8). Bar graph of sodium valproate concentration for physiological saline flow rates of 2.1 and 10 mL/h (*P* = .001). SV = sodium valproate.

The results showed that the SV concentration decreased with an increasing velocity of physiological saline (Fig. 3). The SV concentration in the control group (642.5 ± 23.8 μg/mL) was significantly lower (*P* = .001) than that in the obstruction group (2498.5 ± 51.1 μg/mL; Fig. 4).

### 3.2 In vivo experiment

Before shunt revision, patients’ symptoms included: 3 experiencing confusion; 5, headache and vomiting; and 4, headache and dizziness. Head CT or MRI revealed ventricular enlargement in 8 patients, a slit-like ventricle in 1 patient, and no marked ventricular change in 3 patients. During revision surgery, we observed that all shunts were completely obstructed owing to fracture of the shunt catheter in 2 cases, shunt catheter extrusion in 2, and peritoneal catheter obstruction in 8. Seven days after revision, the symptoms of high intracranial pressure, such as confusion, headache, vomiting, and dizziness disappeared in all patients. CT scans showed that the ventricular volume was decreased in 9 cases and invariant in 3 cases compared with that in the preoperative examination. All patients were discharged from the hospital within 2 weeks after surgery and followed up for 2 months without further indications of malfunction.

**Figure 5:**
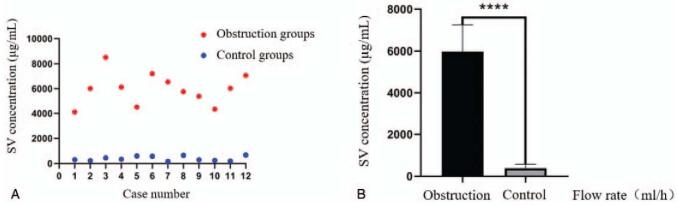
Schematic diagram of in vivo experimental results. (A) Scatter plot of sodium valproate concentration in the cerebrospinal fluid of obstruction and control groups. (B) Histogram of cerebrospinal fluid sodium valproate concentration in the obstruction and control groups (*P* = .001). SV = sodium valproate.

We found that the SV concentration in the malfunction group was significantly higher than that in the control group (5967.8 ± 1 281.3 vs 391.1 ± 184.6 μg/mL, *P* = .001; Fig. 5), and that all SV concentrations in the malfunction group were >2000 μg/mL.

## 4 Discussion

Patients with shunt malfunction can experience symptoms associated with high intracranial pressure, including headaches, vomiting, and even confusion. However, the cause of these symptoms needs to be differentiated from, e.g., cerebral infarction, heat stroke, and electrolyte imbalance. CT and MRI examinations are currently used as reliable methods for diagnosing VP shunt malfunction,^[7,8]^ especially in cases where the ventricular system enlarges again. However, in some patients, the ventricles are fissure-like or not enlarged, and prone to false negatives upon CT and MRI examination. Thus, current diagnostic methods may lead to misdiagnosis, especially in cases of incomplete shunt malfunction. In some patients, only shunt revision surgery is sufficient to confirm malfunction. In this study, 0.1 mL of a 50 mg/mL SV solution was injected into the reservoir. The concentration of SV in the CSF in the capsule was 10 mg/mL. When normal CSF flows through the reservoir, the high SV concentration in the reservoir is continuously diluted.^[9,10]^ In contrast, when the VP shunt malfunctions, the SV in the reservoir cannot be quickly diluted.

According to the principle of material exchange, when the CSF flows through the reservoir, the SV in the reservoir is continuously diluted. We define the initial concentration of SV in the reservoir as *C*_1_, the residual SV concentration detected after 20 minutes as *C*_2_, the volume of the reservoir as *V*_0_, and the volume of CSF flowing through the reservoir as *V*_1_. Under the conditions of full dissolution and exchange, the relationship formula is as follows: *V*_1_ = *V*_0_ × ln(*C*_1_/*C*_2_). In this study, the reservoir was filled with physiological saline with a volume (*V*_0_) of 0.5 mL. After injecting 0.1 mL of a 50 mg/mL SV solution, the initial SV concentration in the reservoir was approximately (*C*_1_) 10 mg/mL. Because of the small volume of the reservoir, full dissolution and exchange of SV and physiological saline is not possible, and there is a certain error between the actual flow rate and the theoretically calculated value. It is necessary to calibrate the flow rate using in vitro experiments, as the in vivo environment is more complicated and requires many experiments for calibration.

Zhang et al^[11]^ reported that VP shunt malfunction can be diagnosed by injecting glucose into the reservoir and detecting the residual concentration. However, the CSF contains different glucose concentrations that can interfere with test results. In this study, we chose an SV solution because it has certain advantages for measurement, such as its good solubility in water while avoiding interference of the CSF itself. In addition, the initial concentration in the reservoir was 10 mg/mL, and the concentration range of SV detected by direct chemiluminescence ranges from 0 to 150 μg/mL. As a result, even if the SV were diluted 10,000 times, it would still be within the detection range, improving detection accuracy. SV is a commonly used antiepileptic drug with a maximum daily dose of 30 mg/kg, and it can be used orally and intravenously. There are no reports on the safety of SV for intraperitoneal injection; however, a 5 mg dose is small and theoretically safe, and none of the patients in the study experienced drug reactions or discomfort during the experiment. However, the possibility of adverse drug reactions requires further investigation.

Broggi et al^[12]^ reported that the velocity of the CSF in the reservoir can be measured by calculating the time for radioactive materials to reach the abdominal cavity when injected into the reservoir. However, this method has not been widely used in clinical practice. Another report showed that cine-phase contrast MRI can be used to detect the velocity of CSF in the tube of the VP shunt for the diagnosis of VP shunt malfunction.^[13]^ However, patient cooperation is required in the examination, and the cost is high; therefore, it has not been widely used in clinical practice.

The VP shunt device used in this study allows for extracorporeal puncture.^[14,15]^ In this study, to avoid damaging the reservoir we used a 1-mL syringe with a 0.45-mm needle for puncturing. We observed no fluid leakage after 200 punctures in in vitro experiments and no CSF extravasation out of the skin after puncture in in vivo experiments, showing that it is a safe method for access to the reservoir. Compared with CT and MRI, this diagnostic method directly reflects the CSF flow. Although it is an invasive procedure, the impact is minimized by the small size of the needle, and none of the patients experienced pain or other complications.

At present, many experts believe that the best velocity of ventricular drainage is approximately 240 mL/d (10 mL/h), and insufficient drainage is defined as a velocity <50 mL/d (2.1 mL/h). Therefore, we defined the control group as a flow rate of 10 mL/h and the obstruction group as that of 2.1 mL/h in in vitro experiments.

According to the results of our in vitro experiments, the lowest SV concentration in patients before malfunction adjustment was 4120.4 μg/mL, the highest concentration in patients with postoperative patency was 668.1 μg/mL, and the average of the 2 was about 2000 μg/mL. Therefore, for diagnosis, we selected a residual SV concentration of 2000 μg/mL as reference value and >2000 μg/mL as PV shunt malfunction. Applying this standard to our data of preoperative malfunction and postoperative patency, the accuracy, sensitivity, and specificity were 100%, indicating the reliability of the results of this method.

However, the diagnostic reliability of our method for patients with insufficient shunts or partial malfunction requires further research. This was a single-center study, and the number of patients recruited was small. A multicenter, randomized controlled study with a large sample size is still needed to further confirm the feasibility and safety of this method.

## 5 Conclusions

The proposed method for the diagnosis of VP shunt malfunction is reliable, safe, and relatively simple, and provides a reference for treatment. Malfunction should be highly suspected when the residual SV concentration is >2000 μg/mL.

## Author contributions

**Conceptualization:** Xinjie Fu, Hongri Zhang.

**Data curation:** Xinjie Fu, Yuhang Chen, Weike Duan, Haixin Yang, Jiulin Xu, Xiaobing Cheng, Hongri Zhang.

**Formal analysis:** Xinjie Fu, Hongri Zhang.

**Funding acquisition:** Hongri Zhang.

**Investigation:** Xinjie Fu, Yuhang Chen, Weike Duan, Haixin Yang, Jiulin Xu, Xiaobing Cheng, Hongri Zhang.

**Methodology:** Xinjie Fu, Yuhang Chen, Weike Duan, Haixin Yang, Jiulin Xu, Xiaobing Cheng, Hongri Zhang.

**Project administration:** Xinjie Fu, Hongri Zhang.

**Resources:** Hongri Zhang.

**Supervision:** Hongri Zhang.

**Writing – original draft:** Xinjie Fu.

**Writing – review & editing:** Hongri Zhang.
